# The General Nursing Council and College of Nursing: A Draft Registration Bill Agreed to

**Published:** 1916-06-17

**Authors:** 


### THE MATRONS' AND SISTERS' DEPARTMENT.

### THE GENERAL NURSING COUNCIL AND COLLEGE OF NURSING.

### A DRAFT REGISTRATION BILL AGREED TO.

LAST week we felt called on to give a clear warning to extremists, if any there were, who might stand out for the Bill, the whole Bill, and nothing but the Bill. It is matter for hearty congratulation that united action has been secured, and the first draft of an agreed Bill carried which provides, by its second clause, that the College of Nursing, Limited, is entitled to drop the word "Limited" and hereafter to be described as the General Nursing Council and College of Nursing. The same clause lays down the democratic basis on which this Council and College will rest after the passing of the Act—that is to say, every nurse registered under this Act will be entitled to vote at elections of the College, and without further fee to become a member of the College of Nursing. All parties are entitled to credit for the agreement they have come to, which cannot fail to have momentous results for good to trained nurses and nursing not only in this country, but throughout the world. We heartily congratulate the Hon. Arthur Stanley upon this result, for its accomplishment could never have been reached but for his able and tactful direction and guidance throughout the whole negotiations. It is indeed a pleasure to note that His Majesty the King has conferred upon Mr. Stanley a Companionship of the Bath.

We publish the full text of the agreed draft Bill for the registration of nurses on page 265. The Bill will be seen to represent most careful drafting, resting upon knowledge and a mastery of the many points which such a Bill had to include. We believe it will meet with the hearty support of trained nurses throughout the United Kingdom. It is matter for congratulation that the Bill, unlike its predecessors, demonstrates that those responsible for it have set their faces against the earlier demands for substantial fees payable by nurses, and that the terms upon which admission to the Register and membership of the College can be obtained are practically nominal. Further, the case of each nurse is to be treated on its merits, and the interests of the nurse and her profession from the outset will be the policy of the General Nursing Council and College of Nursing.

An important matter for nurses and all interested in their welfare is that no time should be lost in writing to the Registrar of the College of Nursing for the necessary papers to apply for registration. The Register is now being formed by the College of Nursing. On page 237 of THE HOSPITAL of the 10th inst. will be found the qualifications for admission to this Register, so that no nurse need experience any difficulty in getting her name placed on the Register without delay.

Last week we published Mr. Stanley's letter of the 7th inst., summoning a meeting of the nominated representatives of the Nurse-Training schools of Hospitals and Poor-Law infirmaries throughout the country, which will be held on the 15th inst. (after the present issue of THE HOSPITAL goes to press) in the Great Hall of St. Thomas's Hospital. This meeting is the most momentous and important one yet held in connection with the organisation and future management of the profession of nursing. Three topics will be discussed and dealt with:—

(1) The draft Bill for the Registration of Nurses, upon which, no doubt, Mr. Stanley will have much of interest to say. We hope he will fully explain the exact bearing of Clause 5 of the draft Bill, which clause defines the basis of appointment of the first Council, which is to hold office for one year. In the absence of such an explanation it would seem to be in conflict with Sub-Section 1 of Clause 4, which provides that not less than two-thirds of the Council shall be elected by the members of the College of Nursing. Is it contemplated that, after the passing of the Act, at the end of the first year there are to be two Councils: (*a*) the General Nursing Council, and (*b*) the Council of the College of Nursing? The importance of this point is manifest.

(2) The meeting will next deal with the formation of the first Register of members of the College and the general conditions for admission to it of nurses now in practice.

(3) The constitution of the Consultative Board; *i.e.*, to decide whether it shall be a large body of 450 to 500 members which at the outset will meet frequently until the whole machinery is successfully built up; and later annually in some important centre in the United Kingdom. In the alternative, it is suggested that there might be a smaller body, say of 100, meeting more frequently to examine in greater detail the proposals formulated by the Consultative Committee. It seems to us at the first blush that it may be well to embody both plans in the scheme by forming all the nominees into (*a*) the General Consultative Board, and (*b*) to elect from their number a smaller body of 100 as its Executive Committee to conduct the examination in greater detail and to prepare and organise the proposed annual Nursing Congress, which will deal with general questions relating to the curriculum, the recognition of nurse-training schools, and other matters of general interest to the nursing profession.

In THE HOSPITAL of May 6, p. 133, Dr. Hurd, the great American authority, stated that nurse registration came originally from England through the late Sir Henry Acland. Nurse registration has not yet been perfected anywhere. Indeed, we make bold to forecast that when the General Nursing Council and College of Nursing is established by Act of Parliament, a perfected system of nurse registration and the strongest and best organisation of trained nurses in the world will speedily be found within the United Kingdom.

